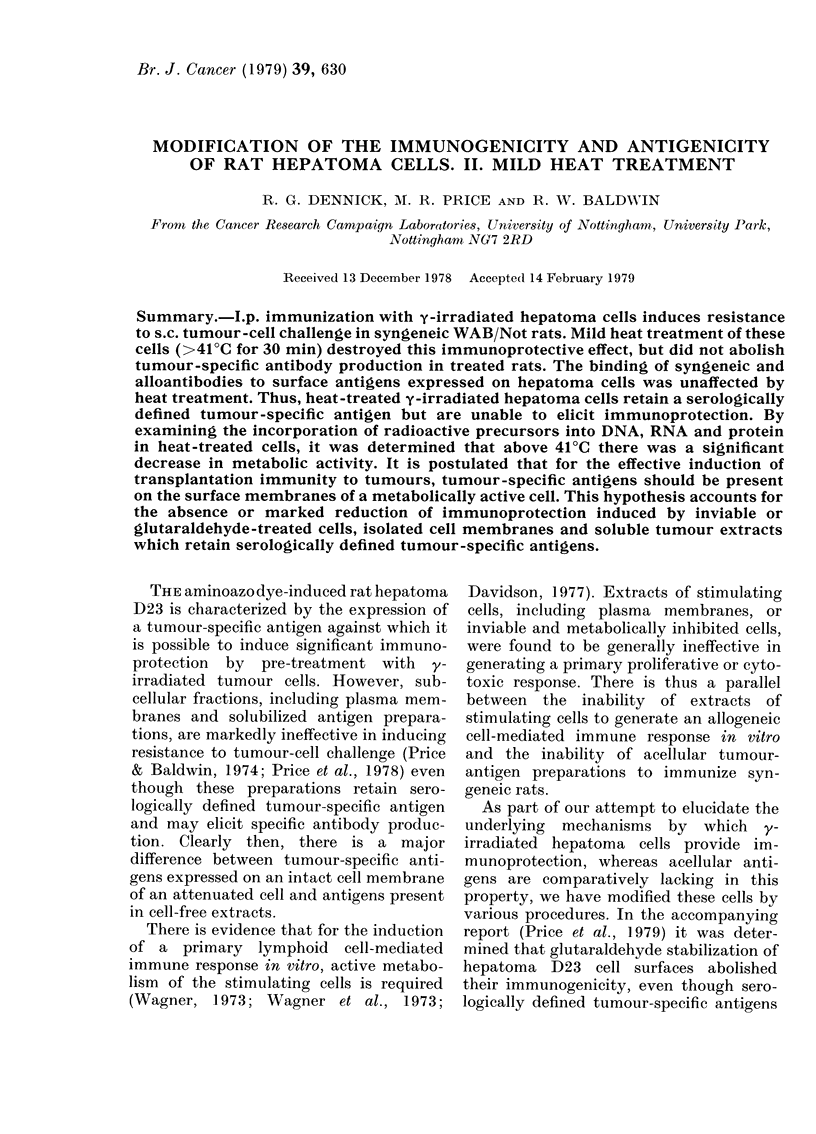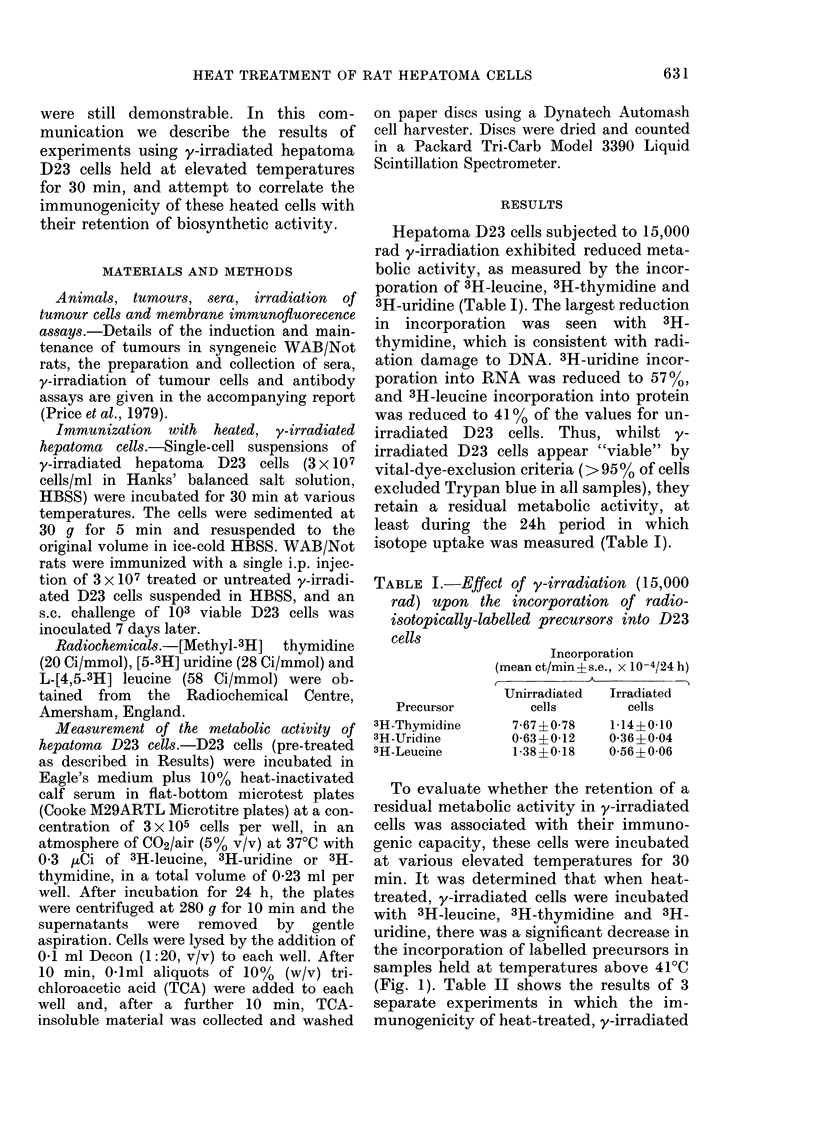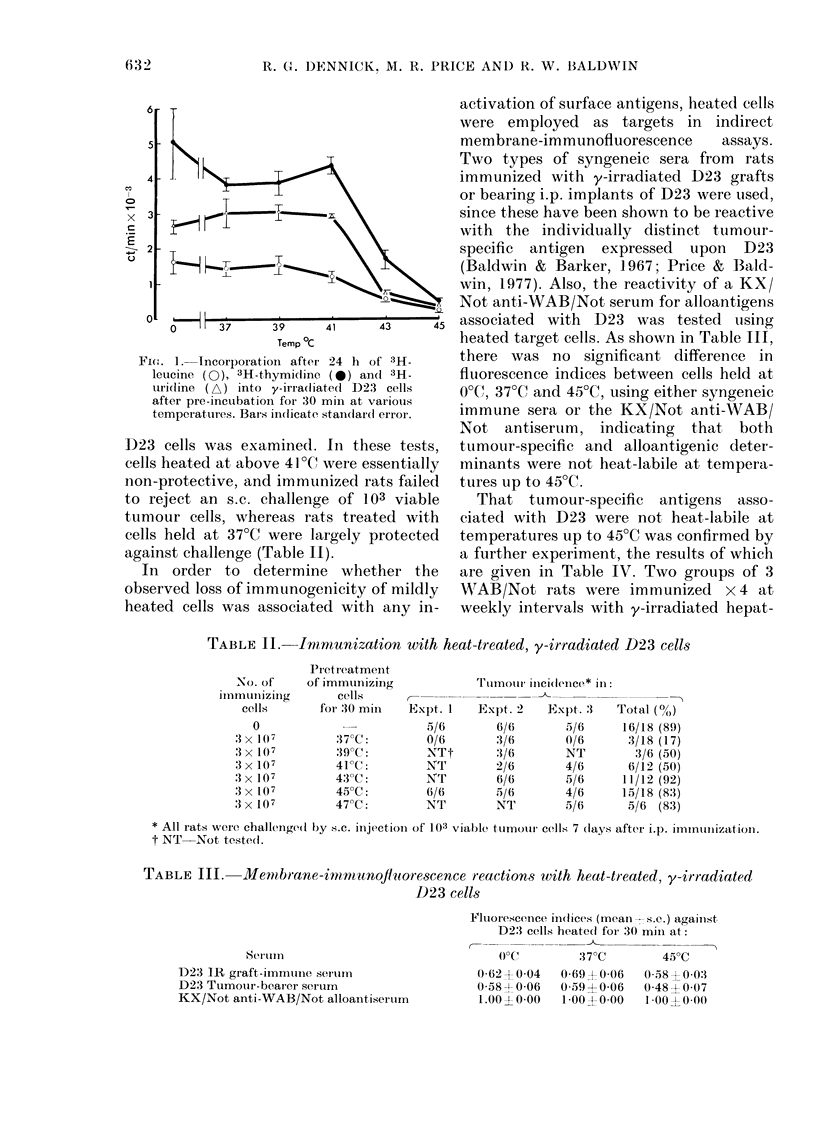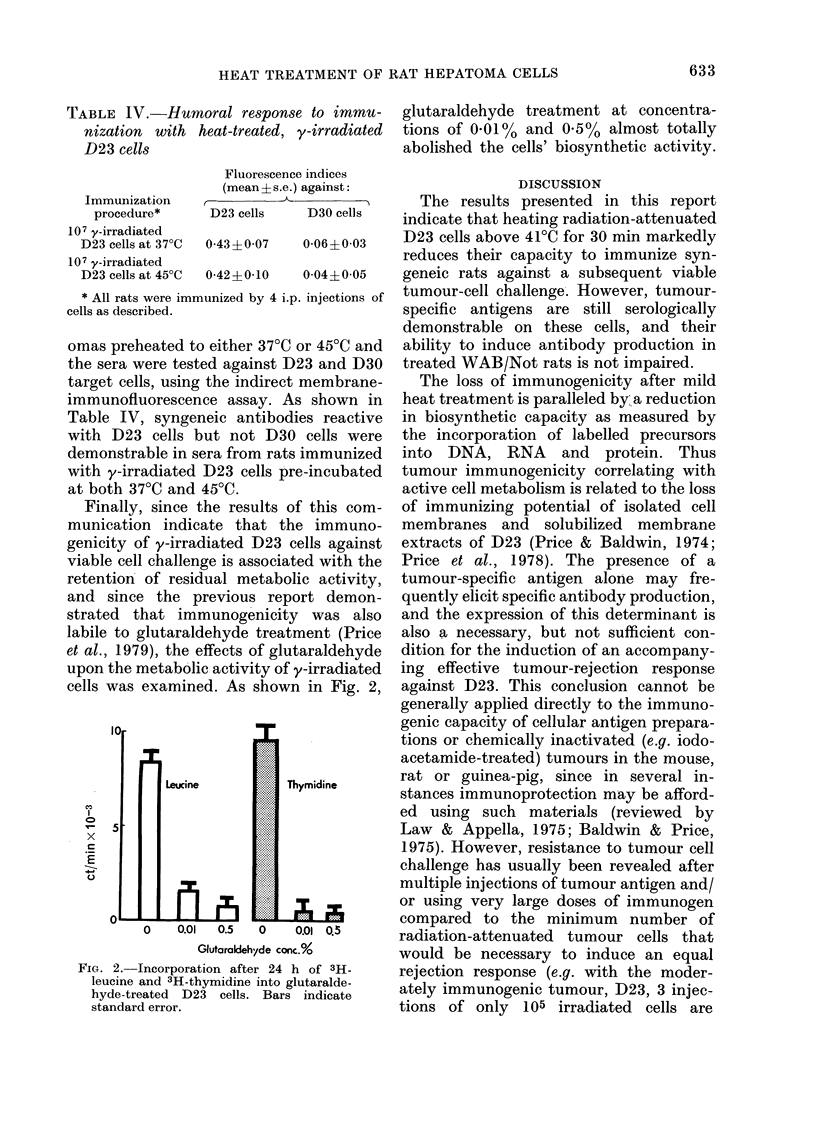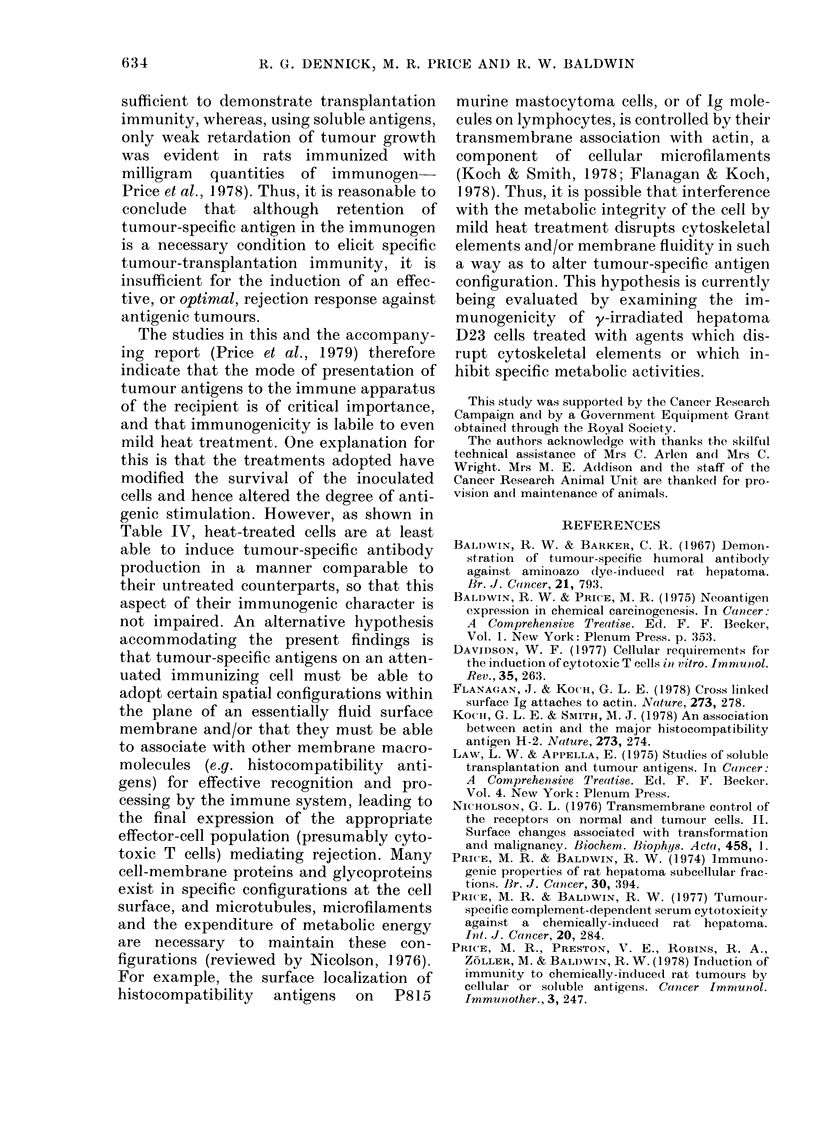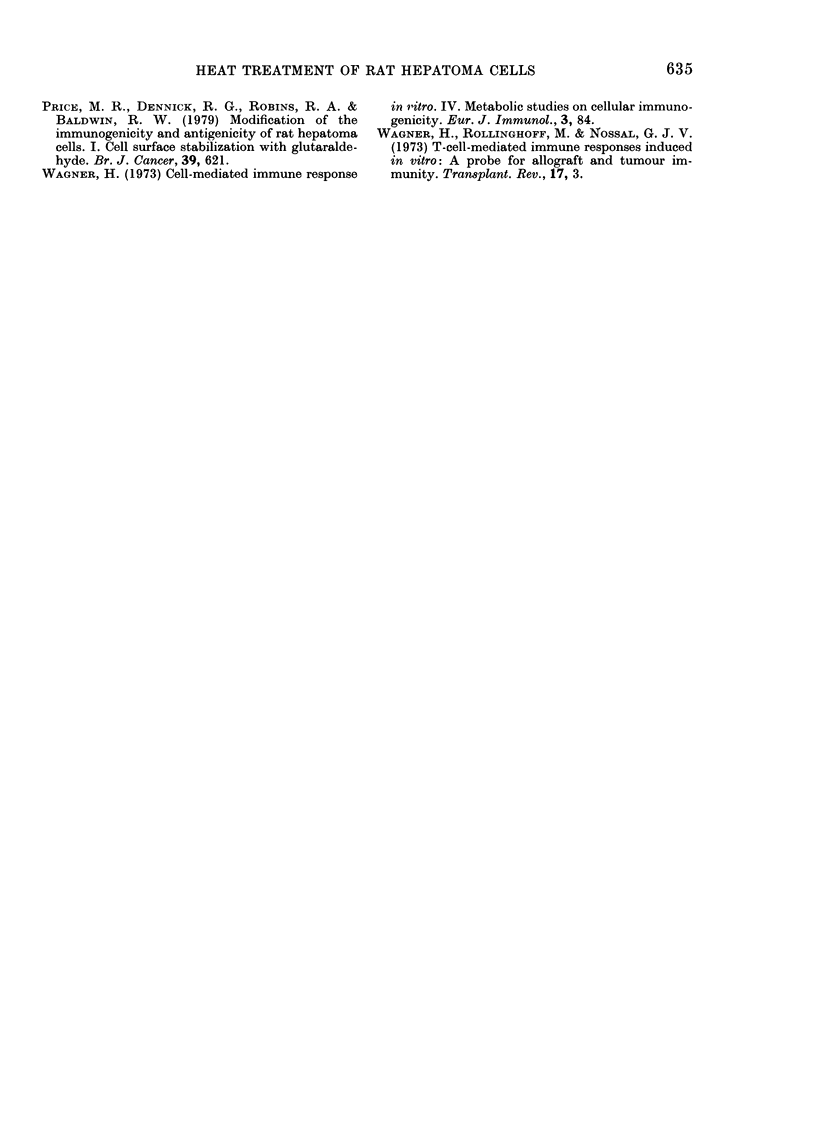# Modification of the immunogenicity and antigenicity of rat hepatoma cells. II. Mild heat treatment.

**DOI:** 10.1038/bjc.1979.114

**Published:** 1979-06

**Authors:** R. G. Dennick, M. R. Price, R. W. Baldwin

## Abstract

I.p. immunization with gamma-irradiated hepatoma cells induces resistance to s.c. tumour-cell challenge in syngeneic WAB/Not rats. Mild heat treatment of these cells (greater than 41 degrees C for 30 min) destroyed this immunoprotective effect, but did not abolish tumour-specific antibody production in treated rats. The binding of syngeneic and alloantibodies to surface antigens expressed on hepatoma cells was unaffected by heat treatment. Thus, heat-treated gamma-irradiated hepatoma cells retain a serologically defined tumour-specific antigen but are unable to elicit immunoprotection. By examining the incorporation of radioactive precursors into DNA, RNA and protein in heat-treated cells, it was determined that above 41 degrees C there was a significant decrease in metabolic activity. It is postulated that for the effective induction of transplantation immunity to tumours, tumour-specific antigens should be present on the surface membranes of a metabolically active cell. This hypothesis accounts for the absence or marked reduction of immunoprotection induced by inviable or glutaraldehyde-treated cells, isolated cell membranes and soluble tumour extracts which retain serologically defined tumour-specific antigens.


*Br. J. Cancer* (1979) **39**, 630

### MODIFICATION OF THE IMMUNOGENICITY AND ANTIGENICITY OF RAT HEPATOMA CELLS. II. MILD HEAT TREATMENT

R. G. DENNICK, M. R. PRICE AND R. W. BALDWIN

*From the Cancer Research Campaign Laboratories, University of Nottingham, University Park, Nottingham NG7 2RD*

Received 13 December 1978 Accepted 14 February 1979

**Summary.—I.p. immunization with γ-irradiated hepatoma cells induces resistance to s.c. tumour-cell challenge in syngeneic WAB/Not rats. Mild heat treatment of these cells (>41°C for 30 min) destroyed this immunoprotective effect, but did not abolish tumour-specific antibody production in treated rats. The binding of syngeneic and alloantibodies to surface antigens expressed on hepatoma cells was unaffected by heat treatment. Thus, heat-treated γ-irradiated hepatoma cells retain a serologically defined tumour-specific antigen but are unable to elicit immunoprotection. By examining the incorporation of radioactive precursors into DNA, RNA and protein in heat-treated cells, it was determined that above 41°C there was a significant decrease in metabolic activity. It is postulated that for the effective induction of transplantation immunity to tumours, tumour-specific antigens should be present on the surface membranes of a metabolically active cell. This hypothesis accounts for the absence or marked reduction of immunoprotection induced by inviable or glutaraldehyde-treated cells, isolated cell membranes and soluble tumour extracts which retain serologically defined tumour-specific antigens.**

THE aminoazo dye-induced rat hepatoma D23 is characterized by the expression of a tumour-specific antigen against which it is possible to induce significant immunoprotection by pre-treatment with γ-irradiated tumour cells. However, subcellular fractions, including plasma membranes and solubilized antigen preparations, are markedly ineffective in inducing resistance to tumour-cell challenge (Price & Baldwin, 1974; Price *et al.*, 1978) even though these preparations retain serologically defined tumour-specific antigen and may elicit specific antibody production. Clearly then, there is a major difference between tumour-specific antigens expressed on an intact cell membrane of an attenuated cell and antigens present in cell-free extracts.

There is evidence that for the induction of a primary lymphoid cell-mediated immune response *in vitro*, active metabolism of the stimulating cells is required (Wagner, 1973; Wagner *et al.*, 1973; Davidson, 1977). Extracts of stimulating cells, including plasma membranes, or inviable and metabolically inhibited cells, were found to be generally ineffective in generating a primary proliferative or cytotoxic response. There is thus a parallel between the inability of extracts of stimulating cells to generate an allogeneic cell-mediated immune response *in vitro* and the inability of acellular tumour-antigen preparations to immunize syngeneic rats.

As part of our attempt to elucidate the underlying mechanisms by which γ-irradiated hepatoma cells provide immunoprotection, whereas acellular antigens are comparatively lacking in this property, we have modified these cells by various procedures. In the accompanying report (Price *et al.*, 1979) it was determined that glutaraldehyde stabilization of hepatoma D23 cell surfaces abolished their immunogenicity, even though serologically defined tumour-specific antigens were still demonstrable. In this communication we describe the results of experiments using $\gamma$-irradiated hepatoma D23 cells held at elevated temperatures for 30 min, and attempt to correlate the immunogenicity of these heated cells with their retention of biosynthetic activity.

### MATERIALS AND METHODS

*Animals, tumours, sera, irradiation of tumour cells and membrane immunofluorecence assays.*—Details of the induction and maintenance of tumours in syngeneic WAB/Not rats, the preparation and collection of sera, $\gamma$-irradiation of tumour cells and antibody assays are given in the accompanying report (Price *et al.*, 1979).

*Immunization with heated, $\gamma$-irradiated hepatoma cells.*—Single-cell suspensions of $\gamma$-irradiated hepatoma D23 cells ($3 \times 10^7$ cells/ml in Hanks' balanced salt solution, HBSS) were incubated for 30 min at various temperatures. The cells were sedimented at 30 *g* for 5 min and resuspended to the original volume in ice-cold HBSS. WAB/Not rats were immunized with a single i.p. injection of $3 \times 10^7$ treated or untreated $\gamma$-irradiated D23 cells suspended in HBSS, and an s.c. challenge of $10^3$ viable D23 cells was inoculated 7 days later.

*Radiochemicals.*—[Methyl-$^3$H] thymidine (20 Ci/mmol), [5-$^3$H] uridine (28 Ci/mmol) and L-[4,5-$^3$H] leucine (58 Ci/mmol) were obtained from the Radiochemical Centre, Amersham, England.

*Measurement of the metabolic activity of hepatoma D23 cells.*—D23 cells (pre-treated as described in Results) were incubated in Eagle's medium plus 10% heat-inactivated calf serum in flat-bottom microtest plates (Cooke M29ARTL Microtitre plates) at a concentration of $3 \times 10^5$ cells per well, in an atmosphere of $CO_2$/air (5% v/v) at 37°C with 0·3 $\mu$Ci of $^3$H-leucine, $^3$H-uridine or $^3$H-thymidine, in a total volume of 0·23 ml per well. After incubation for 24 h, the plates were centrifuged at 280 *g* for 10 min and the supernatants were removed by gentle aspiration. Cells were lysed by the addition of 0·1 ml Decon (1:20, v/v) to each well. After 10 min, 0·1ml aliquots of 10% (w/v) trichloroacetic acid (TCA) were added to each well and, after a further 10 min, TCA-insoluble material was collected and washed on paper discs using a Dynatech Automash cell harvester. Discs were dried and counted in a Packard Tri-Carb Model 3390 Liquid Scintillation Spectrometer.

### RESULTS

Hepatoma D23 cells subjected to 15,000 rad $\gamma$-irradiation exhibited reduced metabolic activity, as measured by the incorporation of $^3$H-leucine, $^3$H-thymidine and $^3$H-uridine (Table I). The largest reduction in incorporation was seen with $^3$H-thymidine, which is consistent with radiation damage to DNA. $^3$H-uridine incorporation into RNA was reduced to 57%, and $^3$H-leucine incorporation into protein was reduced to 41% of the values for unirradiated D23 cells. Thus, whilst $\gamma$-irradiated D23 cells appear "viable" by vital-dye-exclusion criteria (>95% of cells excluded Trypan blue in all samples), they retain a residual metabolic activity, at least during the 24h period in which isotope uptake was measured (Table I).

TABLE I.—*Effect of $\gamma$-irradiation* (15,000 *rad*) *upon the incorporation of radioisotopically-labelled precursors into D*23 *cells*

| | Incorporation (mean ct/min ± s.e., $\times 10^{-4}$/24 h) | |
|---|---|---|
| Precursor | Unirradiated cells | Irradiated cells |
| $^3$H-Thymidine | 7·67 ± 0·78 | 1·14 ± 0·10 |
| $^3$H-Uridine | 0·63 ± 0·12 | 0·36 ± 0·04 |
| $^3$H-Leucine | 1·38 ± 0·18 | 0·56 ± 0·06 |

To evaluate whether the retention of a residual metabolic activity in $\gamma$-irradiated cells was associated with their immunogenic capacity, these cells were incubated at various elevated temperatures for 30 min. It was determined that when heat-treated, $\gamma$-irradiated cells were incubated with $^3$H-leucine, $^3$H-thymidine and $^3$H-uridine, there was a significant decrease in the incorporation of labelled precursors in samples held at temperatures above 41°C (Fig. 1). Table II shows the results of 3 separate experiments in which the immunogenicity of heat-treated, $\gamma$-irradiated

FIG. 1.—Incorporation after 24 h of $^3$H-leucine (○), $^3$H-thymidine (●) and $^3$H-uridine (△) into $\gamma$-irradiated D23 cells after pre-incubation for 30 min at various temperatures. Bars indicate standard error.

D23 cells was examined. In these tests, cells heated at above 41°C were essentially non-protective, and immunized rats failed to reject an s.c. challenge of $10^3$ viable tumour cells, whereas rats treated with cells held at 37°C were largely protected against challenge (Table II).

In order to determine whether the observed loss of immunogenicity of mildly heated cells was associated with any inactivation of surface antigens, heated cells were employed as targets in indirect membrane-immunofluorescence assays. Two types of syngeneic sera from rats immunized with $\gamma$-irradiated D23 grafts or bearing i.p. implants of D23 were used, since these have been shown to be reactive with the individually distinct tumour-specific antigen expressed upon D23 (Baldwin & Barker, 1967; Price & Baldwin, 1977). Also, the reactivity of a KX/Not anti-WAB/Not serum for alloantigens associated with D23 was tested using heated target cells. As shown in Table III, there was no significant difference in fluorescence indices between cells held at 0°C, 37°C and 45°C, using either syngeneic immune sera or the KX/Not anti-WAB/Not antiserum, indicating that both tumour-specific and alloantigenic determinants were not heat-labile at temperatures up to 45°C.

That tumour-specific antigens associated with D23 were not heat-labile at temperatures up to 45°C was confirmed by a further experiment, the results of which are given in Table IV. Two groups of 3 WAB/Not rats were immunized $\times 4$ at weekly intervals with $\gamma$-irradiated hepat-

TABLE II.—*Immunization with heat-treated, $\gamma$-irradiated D23 cells*

| No. of immunizing cells | Pretreatment of immunizing cells for 30 min | Tumour incidence* in: Expt. 1 | Expt. 2 | Expt. 3 | Total (%) |
|---|---|---|---|---|---|
| 0 | — | 5/6 | 6/6 | 5/6 | 16/18 (89) |
| $3 \times 10^7$ | 37°C: | 0/6 | 3/6 | 0/6 | 3/18 (17) |
| $3 \times 10^7$ | 39°C: | NT† | 3/6 | NT | 3/6 (50) |
| $3 \times 10^7$ | 41°C: | NT | 2/6 | 4/6 | 6/12 (50) |
| $3 \times 10^7$ | 43°C: | NT | 6/6 | 5/6 | 11/12 (92) |
| $3 \times 10^7$ | 45°C: | 6/6 | 5/6 | 4/6 | 15/18 (83) |
| $3 \times 10^7$ | 47°C: | NT | NT | 5/6 | 5/6 (83) |

* All rats were challenged by s.c. injection of $10^3$ viable tumour cells 7 days after i.p. immunization.

† NT—Not tested.

TABLE III.—*Membrane-immunofluorescence reactions with heat-treated, $\gamma$-irradiated D23 cells*

| Serum | Fluorescence indices (mean ± s.e.) against D23 cells heated for 30 min at: 0°C | 37°C | 45°C |
|---|---|---|---|
| D23 IR graft-immune serum | $0.62 \pm 0.04$ | $0.69 \pm 0.06$ | $0.58 \pm 0.03$ |
| D23 Tumour-bearer serum | $0.58 \pm 0.06$ | $0.59 \pm 0.06$ | $0.48 \pm 0.07$ |
| KX/Not anti-WAB/Not alloantiserum | $1.00 \pm 0.00$ | $1.00 \pm 0.00$ | $1.00 \pm 0.00$ |

TABLE IV.—*Humoral response to immunization with heat-treated, γ-irradiated D23 cells*

| Immunization procedure* | Fluorescence indices (mean ± s.e.) against: D23 cells | D30 cells |
|---|---|---|
| $10^7$ γ-irradiated D23 cells at 37°C | 0·43 ± 0·07 | 0·06 ± 0·03 |
| $10^7$ γ-irradiated D23 cells at 45°C | 0·42 ± 0·10 | 0·04 ± 0·05 |

* All rats were immunized by 4 i.p. injections of cells as described.

omas preheated to either 37°C or 45°C and the sera were tested against D23 and D30 target cells, using the indirect membrane-immunofluorescence assay. As shown in Table IV, syngeneic antibodies reactive with D23 cells but not D30 cells were demonstrable in sera from rats immunized with γ-irradiated D23 cells pre-incubated at both 37°C and 45°C.

Finally, since the results of this communication indicate that the immunogenicity of γ-irradiated D23 cells against viable cell challenge is associated with the retention of residual metabolic activity, and since the previous report demonstrated that immunogenicity was also labile to glutaraldehyde treatment (Price *et al.*, 1979), the effects of glutaraldehyde upon the metabolic activity of γ-irradiated cells was examined. As shown in Fig. 2,

FIG. 2.—Incorporation after 24 h of $^3$H-leucine and $^3$H-thymidine into glutaraldehyde-treated D23 cells. Bars indicate standard error.

glutaraldehyde treatment at concentrations of 0·01% and 0·5% almost totally abolished the cells' biosynthetic activity.

### DISCUSSION

The results presented in this report indicate that heating radiation-attenuated D23 cells above 41°C for 30 min markedly reduces their capacity to immunize syngeneic rats against a subsequent viable tumour-cell challenge. However, tumour-specific antigens are still serologically demonstrable on these cells, and their ability to induce antibody production in treated WAB/Not rats is not impaired.

The loss of immunogenicity after mild heat treatment is paralleled by a reduction in biosynthetic capacity as measured by the incorporation of labelled precursors into DNA, RNA and protein. Thus tumour immunogenicity correlating with active cell metabolism is related to the loss of immunizing potential of isolated cell membranes and solubilized membrane extracts of D23 (Price & Baldwin, 1974; Price *et al.*, 1978). The presence of a tumour-specific antigen alone may frequently elicit specific antibody production, and the expression of this determinant is also a necessary, but not sufficient condition for the induction of an accompanying effective tumour-rejection response against D23. This conclusion cannot be generally applied directly to the immunogenic capacity of cellular antigen preparations or chemically inactivated (*e.g.* iodoacetamide-treated) tumours in the mouse, rat or guinea-pig, since in several instances immunoprotection may be afforded using such materials (reviewed by Law & Appella, 1975; Baldwin & Price, 1975). However, resistance to tumour cell challenge has usually been revealed after multiple injections of tumour antigen and/or using very large doses of immunogen compared to the minimum number of radiation-attenuated tumour cells that would be necessary to induce an equal rejection response (*e.g.* with the moderately immunogenic tumour, D23, 3 injections of only $10^5$ irradiated cells are sufficient to demonstrate transplantation immunity, whereas, using soluble antigens, only weak retardation of tumour growth was evident in rats immunized with milligram quantities of immunogen—Price *et al.*, 1978). Thus, it is reasonable to conclude that although retention of tumour-specific antigen in the immunogen is a necessary condition to elicit specific tumour-transplantation immunity, it is insufficient for the induction of an effective, or *optimal*, rejection response against antigenic tumours.

The studies in this and the accompanying report (Price *et al.*, 1979) therefore indicate that the mode of presentation of tumour antigens to the immune apparatus of the recipient is of critical importance, and that immunogenicity is labile to even mild heat treatment. One explanation for this is that the treatments adopted have modified the survival of the inoculated cells and hence altered the degree of antigenic stimulation. However, as shown in Table IV, heat-treated cells are at least able to induce tumour-specific antibody production in a manner comparable to their untreated counterparts, so that this aspect of their immunogenic character is not impaired. An alternative hypothesis accommodating the present findings is that tumour-specific antigens on an attenuated immunizing cell must be able to adopt certain spatial configurations within the plane of an essentially fluid surface membrane and/or that they must be able to associate with other membrane macromolecules (*e.g.* histocompatibility antigens) for effective recognition and processing by the immune system, leading to the final expression of the appropriate effector-cell population (presumably cytotoxic T cells) mediating rejection. Many cell-membrane proteins and glycoproteins exist in specific configurations at the cell surface, and microtubules, microfilaments and the expenditure of metabolic energy are necessary to maintain these configurations (reviewed by Nicolson, 1976). For example, the surface localization of histocompatibility antigens on P815 murine mastocytoma cells, or of Ig molecules on lymphocytes, is controlled by their transmembrane association with actin, a component of cellular microfilaments (Koch & Smith, 1978; Flanagan & Koch, 1978). Thus, it is possible that interference with the metabolic integrity of the cell by mild heat treatment disrupts cytoskeletal elements and/or membrane fluidity in such a way as to alter tumour-specific antigen configuration. This hypothesis is currently being evaluated by examining the immunogenicity of $\gamma$-irradiated hepatoma D23 cells treated with agents which disrupt cytoskeletal elements or which inhibit specific metabolic activities.

This study was supported by the Cancer Research Campaign and by a Government Equipment Grant obtained through the Royal Society.

The authors acknowledge with thanks the skilful technical assistance of Mrs C. Arlen and Mrs C. Wright. Mrs M. E. Addison and the staff of the Cancer Research Animal Unit are thanked for provision and maintenance of animals.

### REFERENCES

Baldwin, R. W. & Barker, C. R. (1967) Demonstration of tumour-specific humoral antibody against aminoazo dye-induced rat hepatoma. *Br. J. Cancer*, **21**, 793.

Baldwin, R. W. & Price, M. R. (1975) Neoantigen expression in chemical carcinogenesis. In *Cancer: A Comprehensive Treatise*. Ed. F. F. Becker, Vol. 1. New York: Plenum Press. p. 353.

Davidson, W. F. (1977) Cellular requirements for the induction of cytotoxic T cells *in vitro*. *Immunol. Rev.*, **35**, 263.

Flanagan, J. & Koch, G. L. E. (1978) Cross linked surface Ig attaches to actin. *Nature*, **273**, 278.

Koch, G. L. E. & Smith, M. J. (1978) An association between actin and the major histocompatibility antigen H-2. *Nature*, **273**, 274.

Law, L. W. & Appella, E. (1975) Studies of soluble transplantation and tumour antigens. In *Cancer: A Comprehensive Treatise*. Ed. F. F. Becker. Vol. 4. New York: Plenum Press.

Nicholson, G. L. (1976) Transmembrane control of the receptors on normal and tumour cells. II. Surface changes associated with transformation and malignancy. *Biochem. Biophys. Acta*, **458**, 1.

Price, M. R. & Baldwin, R. W. (1974) Immunogenic properties of rat hepatoma subcellular fractions. *Br. J. Cancer*, **30**, 394.

Price, M. R. & Baldwin, R. W. (1977) Tumour-specific complement-dependent serum cytotoxicity against a chemically-induced rat hepatoma. *Int. J. Cancer*, **20**, 284.

Price, M. R., Preston, V. E., Robins, R. A., Zöller, M. & Baldwin, R. W. (1978) Induction of immunity to chemically-induced rat tumours by cellular or soluble antigens. *Cancer Immunol. Immunother.*, **3**, 247.

PRICE, M. R., DENNICK, R. G., ROBINS, R. A. & BALDWIN, R. W. (1979) Modification of the immunogenicity and antigenicity of rat hepatoma cells. I. Cell surface stabilization with glutaraldehyde. *Br. J. Cancer*, **39**, 621.

WAGNER, H. (1973) Cell-mediated immune response *in vitro*. IV. Metabolic studies on cellular immunogenicity. *Eur. J. Immunol.*, **3**, 84.

WAGNER, H., ROLLINGHOFF, M. & NOSSAL, G. J. V. (1973) T-cell-mediated immune responses induced *in vitro*: A probe for allograft and tumour immunity. *Transplant. Rev.*, **17**, 3.